# Short Musculoskeletal Function Assessment: normative data of the Dutch population

**DOI:** 10.1007/s11136-015-0929-3

**Published:** 2015-02-13

**Authors:** M. W. de Graaf, M. El Moumni, E. Heineman, K. W. Wendt, I. H. F. Reininga

**Affiliations:** 1 Department of Trauma Surgery, University of Groningen, University Medical Center Groningen, P.O. Box 30 001, 9700 RB Groningen, The Netherlands; 2 Department of Surgery, University of Groningen, University Medical Center Groningen, Groningen, The Netherlands

**Keywords:** Short Musculoskeletal Function Assessment, Normative data, Dutch, Netherlands, General population, Patient-reported outcome

## Abstract

**Background:**

The Short Musculoskeletal Function Assessment (SMFA) is widely used in both research and clinical practice. Despite its frequent use, normative data of the SMFA have remained limited. Aim of this study was to gather normative data for the Dutch SMFA (SMFA-NL).

**Methods:**

The SMFA-NL consists of two indices (*function index* and *bother index*) and four subscales (*upper extremity dysfunction*, *lower extremity dysfunction*, *mental and emotional problems*, and *problems with* *daily activities*). A total of 900 patients were invited to fill in the SMFA-NL. Six age groups (18–24, 25–34, 35–44, 45–54, 55–64, and 65–75 years) were constructed. Analysis of variance, *t* tests, and regression analyses were used to assess age and gender effects.

**Results:**

The response rate was 97 %. There was a significant difference between men and women in scores on all indices and subscales (range *p* < 0.001 to *p* = 0.002), except for the *upper extremity dysfunction* subscale (*p* = 0.06). A significant interaction effect was found between gender and age for the *upper extremity dysfunction* subscale; a larger decrease in score with increasing age was observed for women, compared with men. Significant differences were found between age groups for the *bother index* (*p* < 0.001), *lower extremity dysfunction* subscale (*p* = 0.001), and the *problems with daily activities* subscale (*p* = 0.002).

**Conclusion:**

Significant differences in SMFA-NL scores were found between men and women and between different age groups. These SMFA-NL normative data provide an opportunity of benchmarking health status of participants with musculoskeletal disorders or injuries against their age- and gender-matched peers in the Dutch population.

## Introduction

There has been a marked shift internationally in thinking about what health is and how it is measured. Traditional clinical ways of measuring health and the effects of treatment are increasingly accompanied by patient-reported outcome measures (PROMs) [1]. PROMs can be described as an outcome reported directly by patients themselves and not interpreted by an observer. PROMs may include patient assessments of health status, quality of life, satisfaction with care or symptoms, or patient-reported adherence to medication [2]. To date, the use of PROMs is a requirement for clinical trials, funded by the US Food and Drug Administration (FDA) [3]. Since April 2009, the routine collection of PROMs is required by the National Health Services (NHS) in the UK to measure and improve clinical quality of specific elective surgical procedures, such as hip and knee replacement and inguinal hernia repair [4, 5].

The challenge for clinicians is to interpret the meaningfulness of the scores, derived from PROMs. Some additional information is necessary to place changes in scores, and scores for different ages and gender, within a clinical context [6]. Traditionally, comparisons are made between pre- and post-treatment data to determine whether a treatment is responsible for change in functioning, relative to a control or comparison group [7]. However, in studies looking at acute onset conditions (e.g., injury), participants are often recruited after the health event has taken place. In this case, participants can be asked to “recall” their pre-onset health status [8]. Therefore, in studies regarding injured patients, retrospective measurements of pre-injury health status are often used for reference values [9–11]. However, these retrospective measurements may be subject to recall bias. When one is interested in clinical significance, Kendall et al. raised two basic questions: (a) Is the amount of change that has occurred, large enough to be considered meaningful and (b) Are treated individuals distinguishable from “normal individuals” serving as a reference group [7]? The first question regards the clinimetric properties of a questionnaire, such as the minimally important change (MIC) and the smallest detectable change (SDC) [12]. Normative comparisons address the second question. Specifically, a normative comparison addresses the issues of severity. Normative data can be used to assess whether patients treated for specific conditions have returned to or at least have come closer to their normative ranges of functioning [6, 13]. Furthermore, normative comparison allows the assessment of the effectiveness of a treatment against a standard independent of the patients at issue [7]. Additionally, the absence of population-based normative values may be a barrier to the routine use of patient-reported outcome measures (PROMs) in clinical practice, because of the challenge of interpreting scores at the individual patient level [14, 15]. In order to enhance multinational comparisons, PROMs need to be available in multiple languages, and normative data of these PROMs for different countries are needed.

The Short Musculoskeletal Function Assessment (SMFA) is a widely used PROM to assess physical functioning of patients with a variety of musculoskeletal disorders [16]. The SMFA has been cross-culturally adapted to various languages [17–23]. The SMFA can be used to assess and compare different types of musculoskeletal injuries and disorders. The SMFA is recommended by the American Academy of Orthopedic Surgeons (AAOS) for use in clinical practice to assess the effectiveness of treatment regimens and in musculoskeletal research settings to study the clinical outcomes of treatment [24]. Hence, the SMFA is widely used in clinical research on musculoskeletal injuries of the upper and lower extremities [25–27]. The normative values for the SMFA in the general population of the USA have been published [6, 28]. Recently, the SMFA has been translated and cross-culturally adapted into Dutch (SMFA-NL) [20]. The SMFA-NL was found to be a valid and reliable PROM. However, normative values of the SMFA-NL are not yet established for the Dutch population. The aim of this study was therefore to gather normative data of the SMFA-NL in a general Dutch population sample.

## Materials and methods

### Questionnaire

The original American SMFA consists of 46 items ordered as two indices: the *function index* (34 items) and the *bother index* (12 items) [16]. The Dutch version of the SMFA (SMFA-NL) [20] can be divided, next to the two indices, into four subscales: the *upper extremity dysfunction* (6 items), *lower extremity dysfunction* (12 items), *problems with daily activities* (20 items), and *mental and emotional problem* (8 item) subscales. All items are scored on a 5-item Likert scale, ranging from 1 (good function/not bothered) to 5 (poor function/extremely bothered). Total scores on the subscales are calculated by summing the responses to the individual items and transforming the scores so that the range is from 0 to 100, with higher scores indicating poorer function [16, 20].

Next to basic demographic characteristics, such as age and gender, information on marital status, educational level, and current health problems were obtained. Current health problems (within the previous 6 months) were obtained by means of a questionnaire, comprising 12 chronic health conditions. This questionnaire is developed by the Dutch National Institute for Public Health and the Environment to assess health conditions of the Dutch population [29]. A Web-based questionnaire was developed, containing the SMFA-NL and questions regarding demographic characteristics and chronic health conditions.

### Sample and data collection

A population-based sample was randomly chosen from the database of respondents of an independent marketing firm, which contained postal codes from all provinces of the Netherlands. Stratified random sampling was used to avoid bias because of gender and age differences. Furthermore, the aim was to obtain a fixed precision for each age and gender group estimate rather than to represent the demographics of the entire Dutch population. Six age groups (18–24, 25–34, 35–44, 45–54, 55–64, and 65–75 years) were constructed. Of these age groups, 50 % of the approached participants were women. Previous research with the SMFA-NL has shown a response rate of around 65 % [6, 20]. Hence, in order to achieve a sample size of 100 participants per age group, a total sample of 900 persons was recruited. The sample was recruited by e-mail, in which the purpose of the study was explained, and it contained a link to the Web site with the electronic questionnaire. Non-responders were reminded once.

### Statistical analysis

Descriptive statistics, including means, standard deviations, and 95 % confidence intervals, were calculated to present the normative data. Data of the subscales were transposed, so that higher scores indicated better function. To determine the internal consistency of the American indices and the four Dutch subscales of the SMFA-NL, Cronbach’s alpha was calculated. It is widely accepted that Cronbach’s alpha should be between 0.70 and 0.95 [12].

Differences between men and women were tested with independent *t* tests. To assess differences between age groups, an analysis of variance (ANOVA) with Bonferroni post hoc analysis was performed. Multiple linear regression analysis was used to analyze whether there was a significant interaction between gender and age groups. Cases that contained one or more missing items in an index or subscale of the SMFA-NL were excluded from further analysis for that certain subscale. Statistical analyses were performed using IBM SPSS Statistics for Windows, version 20.0. Armonk, NY: IBM Corp.

## Results

Eight hundred and seventy-five subjects (97 %) responded to the questionnaire. The demographic characteristics of the respondents are presented in Table 1. Gender was equally distributed among the age groups. All of the 875 respondents reported whether they had a chronic health condition (Table 2). Of the 875 returned questionnaires, 623 (71 %) did not have missing items, 163 (19 %) of the questionnaires had one missing item, 49 (6 %) had two missing items, and 40 (5 %) had three or more missing items. Of the 46 items of the SMFA, item 15 and 22 were missing in 7.8 and 6.7 % of all, cases respectively (Table 3). These were items regarding driving a vehicle and sexual activity. Excellent Cronbach’s alpha values (≥0.87) were obtained for all subscales and indices (Table 4).

**Table 1:** Demographic characteristics of the respondents

Demographics	*N* (%)
Age (years) (*N* = 864)	
18–24	146 (17)
25–34	141 (16)
35–44	148 (17)
45–54	138 (16)
55–64	143 (17)
65–75	148 (17)
Gender (*N* = 840)	
Male	420 (50)
Female	420 (50)
Marital status (*N* = 811)	
Single	220 (27)
With partner	322 (40)
With partner and children	236 (29)
With children	33 (4)
Educational level (*N* = 864)	
Elementary school	22 (3)
High school	307 (35)
College	268 (31)
Bachelor’s degree or higher	267 (31)

**Table 2:** Reported chronic health conditions per age group

	Age groups^a^					
	18–24	25–34	35–44	45–54	55–64	65–75
*Chronic health condition*						
Migraine	54 (37)	36 (26)	33 (22)	44 (32)	25 (18)	22 (15)
Hypertension	10 (7)	10 (7)	13 (9)	35 (25)	59 (41)	63 (43)
Osteoarthritis	4 (3)	5 (4)	15 (10)	19 (14)	48 (34)	64 (44)
Asthma, chronic bronchitis, lung emphysema	22 (15)	13 (9)	23 (16)	15 (11)	25 (18)	16 (11)
Severe spinal disease, including disc hernia	8 (6)	9 (6)	19 (13)	23 (17)	21 (15)	30 (20)
Inflammatory bowel disease	11 (8)	12 (9)	14 (10)	9 (7)	9 (6)	8 (5)
Rheumatoid arthritis	4 (3)	8 (6)	11 (7)	11 (8)	9 (6)	20 (14)
Diabetes mellitus	2 (1)	3 (2)	3 (2)	8 (6)	15 (12)	33 (23)
Heart failure	2(1)	2 (1)	2 (1)	5 (4)	13 (9)	11 (7)
Myocardial infarction or angina pectoris	2 (1)	1 (1)	1 (1)	1 (1)	7 (5)	14 (10)
Cancer	1 (1)	1 (1)	4 (3)	2 (1)	6 (4)	9 (6)
Stroke	0 (0)	2 (1)	2 (1)	4 (3)	3 (2)	5 (3)
*Number of chronic health conditions*						
None	70 (48)	75 (53)	69 (47)	49 (36)	38 (27)	31 (21)
One	45 (31)	45 (32)	47 (32)	44 (32)	40 (28)	36 (24)
Two	23 (16)	13 (9)	16 (11)	22 (16)	34 (24)	38 (26)
Three or more	8 (5)	8 (6)	16 (11)	23 (17)	31 (22)	43 (29)

^a^Data are presented as *N* (%)

**Table 3:** Number of missing values per item of the SMFA-NL

Number of missing values per item																
Item number	1	2	3	4	5	6	7	8	9	10	11	12	13	14	15	16
Not answered	0	7	3	7	8	4	2	6	8	9	14	13	13	14	68	14
% not answered	0.0	0.8	0.3	0.8	0.9	0.5	0.2	0.7	0.9	1.0	1.6	1.5	1.5	1.6	7.8	1.6

Item number	17	18	19	20	21	22	23	24	25	26	27	28	29	30	31	32
Not answered	8	6	9	5	6	59	8	4	32	11	22	14	11	11	10	6
% not answered	0.9	0.7	1.0	0.6	0.7	6.7	0.9	0.5	3.7	1.3	2.5	1.6	1.3	1.3	1.1	0.7

Item number	33	34	35	36	37	38	39	40	41	42	43	44	45	46		
Not answered	10	7	3	4	3	4	6	7	8	6	9	5	7	5		
% not answered	1.1	0.8	0.3	0.5	0.3	0.5	0.7	0.8	0.9	0.7	1.0	0.6	0.8	0.6

**Table 4:** Internal consistency of the SMFA-NL

	*n*	No. of items	Cronbach’s alpha
*Indices*			
Function Index	643	34	0.96
Bother Index	832	12	0.94
*Subscales*			
Upper extremity dysfunction	842	6	0.91
Lower extremity dysfunction	751	12	0.92
Problems with daily activities	717	20	0.97
Mental and emotional problems	841	8	0.87

Statistically significant differences in SMFA-NL scores were found between men and women on all indices and subscales (ranging from *p* < 0.001 to *p* = 0.002), except for the *upper extremity dysfunction* subscale (*p* = 0.06). A significant interaction was found between gender and age groups for the *upper extremity dysfunction* subscale (*p* = 0.03), indicating that the effect of aging on the upper extremity function is different for men and women. No interaction effects were found for the other subscales and indices. These analyses were not adjusted for other demographic characteristics.

Significant differences in scores on the *bother index* and two subscales were found between various age groups. With regard to the *bother index*, significant differences were found between age groups 18–24 and 45–54 (*p* = 0.03), 18–24 and 55–64 (*p* < 0.01), and 18–24 and 64–75 (*p* = 0.03). For the *lower extremity dysfunction* subscale, significant differences were seen between age groups 18–24 and 65–74 (*p* = 0.03), 25–34 and 55–64 (*p* = 0.05), and 25–34 and 65–74 (*p* = 0.01). For the *problems with daily activities* subscale, significant differences were found between age groups 18–24 and 55–64 (*p* = 0.01), and 25–34 and 55–64 (*p* = 0.04). No significant differences in scores on the *function index* (*p* = 0.22), the *upper extremity dysfunction* subscale (*p* = 0.09), and *mental and emotional problems* subscale (*p* = 0.83) were observed between the age groups.

In Table 5, the normative data of the SMFA-NL are presented. Mean scores of the *function index* and *bother index* were 88.5 (SD 13.2) and 87.0 (SD 17.5), respectively. Mean scores of the *lower extremity dysfunction, problems with daily activities,* and *mental and emotional problems* were 89.9 (SD 14.2), 87.8 (SD 17.7), and 78.7 (SD 17.2), respectively. Because of the interaction effect between age and gender for the *upper extremity dysfunction* subscale, age- and gender-specific normative data are presented for this subscale. Mean score for the *upper extremity dysfunction* subscale was 96.5 (SD 9.3) for men and 93.8 (SD 13.6) and for women.

**Table 5:** Normative data for the SMFA-NL

	*N*	Mean (SD)	95% CI
*Indices*			
Function Index			
18–24	121	90.0 (12.2)	87.8–92.2
25–34	112	89.8 (12.7)	87.4–92.2
35–44	113	89.3 (13.3)	86.8–91.8
45–54	98	87.6 (14.1)	84.7–90.4
55–64	106	86.5 (12.7)	84.1–89.0
65–75	83	87.1 (13.5)	84.1–90.0
Males	321	90.2 (11.2)	88.9–91.4
Females	296	86.6 (14.7)	84.9–88.3
Total	643	88.5 (13.2)	87.4–89.5
Bother index			
18–24	141	91.3 (14.2)	88.9–93.6
25–34	137	90.6 (14.8)	88.1–93.1
35–44	142	87.0 (19.3)	83.8–90.2
45–54	135	84.6 (18.6)	81.5–87.8
55–64	134	83.1 (17.7)	80.1–86.1
65–75	133	84.7 (18.7)	81.5–87.9
Males	401	88.8 (15.2)	87.3–90.3
Females	398	84.7 (19.5)	82.8–86.7
Total	832	87.0 (17.5)	85.8–88.2
*Subscales*			
Upper extremity dysfunction			
Males			
18–24	47	98.4 (7.6)	96.2–100.0
25–34	53	96.2 (12.2)	92.9–99.6
35–44	57	96.8 (9.2)	94.3–99.2
45–54	76	96.9 (7.2)	95.3–98.6
55–64	86	95.4 (9.2)	93.5–97.5
65–75	83	96.1 (10.1)	93.9–98.3
Total	403	96.5 (9.3)	95.6–97.4
Females			
18–24	87	95.8 (10.1)	93.7–98.0
25–34	75	96.6 (10.3)	94.3–99.1
35–44	81	94.1 (15.0)	90.9–97.5
45–54	57	94.2 (13.8)	90.6–97.9
55–64	49	89.2 (13.7)	85.3–93.2
65–75	52	89.5 (17.5)	84.6–94.4
Total	405	93.8 (13.6)	95.4–95.1
Lower extremity dysfunction			
18–24	131	92.0 (13.5)	89.6–94.3
25–34	126	92.9 (12.3)	90.7–95.1
35–44	135	90.9 (14.6)	88.4–93.4
45–54	117	89.0 (14.7)	86.3–81.7
55–64	120	87.6 (13.8)	85.1–90.1
65–75	112	86.4 (14.8)	83.6–89.1
Males	369	90.9 (12.8)	90.9–92.2
Females	353	88.8 (15.4)	87.2–90.4
Total	751	89.9 (14.2)	88.8–90.9
Problems with daily activities			
18–24	132	91.4 (14.5)	88.6–93.9
25–34	124	90.5 (15.4)	87.7–93.2
35–44	123	88.7 (16.9)	85.7–91.7
45–54	112	85.2 (19.6)	81.5–88.8
55–64	115	83.9 (17.9)	80.6–87.2
65–75	100	86.0 (17.3)	82.6–89.4
Males	357	89.6 (14.7)	89.6–91.1
Females	329	85.5 (19.3)	83.4–87.6
Total	717	87.8 (17.1)	86.5–89.0
Mental and emotional problems			
18–24	141	78.5 (16.0)	75.9–81.2
25–34	138	79.5 (17.2)	76.6–82.4
35–44	144	77.8 (20.1)	74.5–81.1
45–54	133	78.1 (17.6)	75.1–81.2
55–64	138	78.0 (15.3)	75.4–80.6
65–75	137	80.2 (17.1)	77.3–83.1
Males	407	81.9 (15.1)	80.4–83.4
Females	401	75.2 (18.7)	73.3–77.0
Total	841	78.7 (17.2)	77.6–79.9

Age- and gender-specific normative data for all indices and subscales are presented in “Appendix.”

## Discussion

The definition of health is changing. WHO defined it in 1948 as “a state of complete physical, mental and social well-being and not merely the absence of disease or infirmity.” Recently, Huber et al. [30] proposed changing the emphasis toward the ability to adapt and self-manage in the face of social, physical, and emotional challenges. Patients’ perspective regarding their health status is gaining popularity in clinical research as well as in daily clinical practice. PROMS are increasingly used to capture these perspectives.

The SMFA was developed as an instrument to assess physical functioning of patients with a variety of musculoskeletal disorders [16]. Aim of this study was to gather normative data of the SMFA-NL of the Dutch population. Normative data are essential in the process of exploring the gap between patients with musculoskeletal injuries and the general population, determining therapeutic effectiveness or whether a patient has recovered to an acceptable level of functioning. Yet, it is not clear whether the general population scores are representative for specific subsets of the general population, like trauma patients. However, the general population scores represent an acceptable level of functioning for those who are recovering from an injury or disease.

Internal consistency of the two indices and four subscales of the SMFA-NL were high, with Cronbach’s alpha values exceeding 0.87. These results are in line with previous studies regarding the clinimetric properties of the SMFA. Swiontkowski et al. [16] reported high internal consistency measures (0.95) for both the *function index* and *bother index* of the original SMFA questionnaire. Reininga et al. [20] reported high Cronbach’s alpha values (>0.85) for the SMFA-NL. These values were comparable to other cross-cultural adaptation studies of the SMFA as well [18, 21–23].

Little normative data of the SMFA have been published. Hunsaker et al. [6] reported normative data for the general population of the USA. The normative data for the *function index* and the *bother index* found in this study are comparable to the normative data found by Hunsaker et al. [6]. However, Hunsaker et al. [6] have not presented normative data for men and women separately, nor for different age groups. These have been presented by Barei et al. [28], but only for the *function index.*

In this study, significant differences in SMFA-NL scores between men and women were found for both indices and all subscales, except for the *upper extremity dysfunction* subscale. Barei et al. [28] also reported significant differences in normative values of the *function index* between men and women. The difference in scores of the *upper extremity dysfunction* subscale between men and women was borderline significant (*p* = 0.06).

The present study showed significant differences between age groups in scores on the *bother index*, and the *lower extremity dysfunction* and *problems with daily activities* subscales. Further exploration showed that these results were due to differences between the two youngest (18–24 and 25–34) and the two eldest (55–64 and 65–75) age groups. These differences in SMFA-NL scores might be due to aging.

A significant interaction effect was found in this study between gender and age for the *upper extremity dysfunction* subscale. Scores on the *upper extremity dysfunction* subscale showed a larger decrease in score with increasing age for women, compared to men. Differences in SMFA scores between age groups or an interaction effect between gender and age have not been reported in previous studies on normative values of the SMFA. This same kind of interaction between age and gender has been found in normative data of the Disabilities of Arm, Shoulder and Hand (DASH) PROM [31].

Interpretability (e.g., the clinical relevance) of the (differences in) scores on the indices and subscales of the SMFA-NL remains difficult, since a minimal important change (MIC) score has not yet been established. Hence, further research into the interpretability of the SMFA, such as the minimally important change, is needed.

Some strong points and limitations of this study have to be addressed. First of all, the response rate in this study was high (97 %), and overall, the number of missing values was low. Questions regarding driving a vehicle and sexual activity had the most missing values (7.8 and 6.7 %, respectively). Both questions were more often left unanswered by elderly. Additionally, the sample size of the age- and gender-specific normative data might be seen as a possible limitation. However, the 95 % confidence intervals of the age- and gender-specific normative data were narrow. Additionally, because the sampling of this study was gender- and age group specific, demographic data of the total study population were slightly different from the total Dutch population. However, the study sample was randomly drawn from a large database that contained postal codes of all Dutch provinces. Overall, the prevalence of reported chronic diseases and multimorbidity in the present study were slightly higher compared with other data reported for the Dutch population [33]. However, these data for the Dutch population were based on data provided by Dutch general practitioners. Several studies have shown that, when using self-reported questionnaires, the prevalence of chronic diseases is higher compared with data reported by the general practitioner [34, 35]. However, per age group, demographic data were considered to be representative for the Dutch population [32].

In conclusion, this study provides normative data for the Dutch SMFA (SMFA-NL). The normative values were comparable to previously published normative data of the SMFA. Significant differences in SMFA-NL scores were found between men and women and between different age groups, which stresses the importance of presenting age- and gender-specific normative values of the SMFA-NL. These SMFA-NL normative values provide an opportunity of benchmarking health status of participants with musculoskeletal disorders or injuries against their age- and gender-matched peers in the Dutch population. Whether these values are representative for specific subsamples of the general population, for example trauma patients, has to be established.

## Appendix: Age- and gender-specific normative data for the SMFA-NL

**Table:**

	*N*	Mean (SD)	95% CI		*N*	Mean (SD)	95% CI
*Indices*							
Function Index							
Males				Females			
18–24	38	93.8 (8.2)	91.1–96.5	18–24	78	88.4 (13.0)	84.5–91.3
25–34	46	91.0 (14.2)	86.7–95.2	25–34	62	88.6 (11.9)	85.6–91.6
35–44	50	91.1 (11.6)	87.6–94.4	35–44	61	87.7 (14.6)	83.9–91.4
45–54	63	88.0 (13.6)	84.6–91.5	45–54	33	87.0 (15.6)	81.5–92.5
55–64	66	89.1 (9.7)	86.8–91.5	55–64	36	82.0 (15.4)	76.8–87.2
65–75	57	89.8 (8.1)	87.7–91.9	65–75	22	79.6 (21.2)	70.2–89.0
Total	321	90.2 (11.2)	88.9–91.4	Total	296	86.6 (14.7)	84.9–88.3
Bother Index							
Males				Females			
18–24	46	94.1 (10.1)	91.1–97.1	18–24	91	89.5 (16.0)	86.2–92.8
25–34	54	93.1 (13.8)	89.3–96.9	25–34	75	88.0 (15.7)	84.4–91.6
35–44	62	91.1 (14.2)	87.5–94.7	35–44	78	83.5 (22.2)	78.5–88.5
45–54	74	86.3 (17.2)	82.3–90.3	45–54	57	82.5 (20.5)	77.1–88.0
55–64	84	86.1 (15.3)	82.8–89.4	55–64	46	77.9 (19.9)	72.0–83.8
65–75	80	86.1 (15.8)	82.6–89.7	65–75	48	81.3 (22.9)	74.7–88.0
Total	401	88.8 (15.2)	87.3–90.3	Total	398	84.7 (19.5)	82.8–86.7
*Subscales*							
Upper extremity dysfunction							
Males				Females			
18–24	47	98.4 (7.6)	96.2–100.0	18–24	87	95.8 (10.1)	93.7–98.0
25–34	53	96.2 (12.2)	92.9–99.6	25–34	75	96.6 (10.3)	94.3–99.1
35–44	57	96.8 (9.2)	94.3–99.2	35–44	81	94.1 (15.0)	90.9–97.5
45–54	76	96.9 (7.2)	95.3–98.6	45–54	57	94.2 (13.8)	90.6–97.9
55–64	86	95.4 (9.2)	93.5–97.5	55–64	49	89.2 (13.7)	85.3–93.2
65–75	83	96.1 (10.1)	93.9–98.3	65–75	52	89.5 (17.5)	84.6–94.4
Total	403	96.5 (9.3)	95.6–97.4	Total	405	93.8 (13.6)	95.4–95.1
Lower extremity dysfunction							
Males				Females			
18–24	40	96.4 (8.1)	93.7–99.0	18–24	86	90.1 (14.5)	87.0–93.2
25–34	50	92.6 (14.3)	88.5–96.6	25–34	70	92.9 (11.1)	90.2–95.5
35–44	59	93.4 (11.6)	90.4–96.4	35–44	74	88.8 (16.6)	84.9–92.6
45–54	68	89.0 (15.8)	85.1–92.8	45–54	47	89.0 (13.5)	85.0–92.9
55–64	77	89.7 (10.8)	87.2–92.1	55–64	39	83.9 (17.4)	78.2–89.5
65–75	74	87.5 (12.3)	84.6–90.4	65–75	33	83.0 (19.5)	76.1–89.9
Total	369	90.9 (12.8)	89.5–92.2	Total	353	88.8 (15.4)	87.2–15.4
Problems with daily activities							
Males				Females			
18–24	44	94.3 (10.4)	91.2–97.5	18–24	84	89.5 (16.4)	86.0–93.1
25–34	50	92.3 (15.8)	87.8–96.8	25–34	67	88.6 (15.7)	84.8–92.4
35–44	57	91.3 (13.2)	87.8–94.4	35–44	64	86.3 (19.6)	81.4–91.2
45–54	68	86.7 (17.6)	82.5–91.0	45–54	40	82.4 (22.9)	75.0–89.7
55–64	72	87.0 (14.9)	83.5–90.5	55–64	39	78.5 (20.6)	71.8–85.2
65–75	65	88.4 (13.4)	85.1–91.8	65–75	31	79.4 (23.0)	71.0–87.9
Total	357	89.6 (14.7)	88.0–91.1	Total	329	85.5 (19.3)	19.3–87.6
Mental and emotional problems							
Males				Females			
18–24	47	83.1 (12.8)	79.3–86.9	18–24	90	75.9 (17.1)	72.4–79.5
25–34	54	83.6 (14.7)	79.6–87.6	25–34	77	75.7 (18.4)	71.5–79.9
35–44	62	83.1 (17.5)	87.7–87.6	35–44	80	73.4 (21.0)	68.7–78.0
45–54	73	79.4 (16.9)	75.5–83.3	45–54	56	76.8 (18.5)	71.8–81.7
55–64	86	80.3 (14.1)	77.3–83.4	55–64	48	73.8 (16.3)	69.0–78.5
65–75	84	82.8 (13.9)	79.8–85.8	65–75	47	75.1 (21.1)	86.9–81.3
Total	407	81.9 (15.1)	80.4–83.4	Total	401	75.2 (18.7)	73.3–77.0
